# Neuropathology studies of dementia in US persons other than non-Hispanic whites

**DOI:** 10.17879/freeneuropathology-2022-3795

**Published:** 2022-03-10

**Authors:** My-le Nguyen, Emily Z. Huie, Rachel A. Whitmer, Kristen M. George, Brittany N. Dugger

**Affiliations:** 1 Department of Pathology and Laboratory Medicine, University of California, Davis USA; 2 Department of Public Health Sciences, University of California, Davis USA

**Keywords:** Disparities, Dementia, Neuropathology, Hispanic, Latino, Asian, African American, Brain

## Abstract

Alzheimer’s disease (AD) and vascular dementia are two of the most prevalent dementias that afflict the aging population in the United States (US). Studies have made great strides in understanding the neuropathology of these diseases; however, many studies are conducted in the context of non-Hispanic whites (NHWs), and few include the rapidly growing underrepresented populations that reside in the US. We sought to characterize current knowledge of the neuropathologic landscape of AD and vascular dementia of the largest growing US minority groups, namely Latinos/Hispanics, Black Americans, and Asian Americans, compared with NHWs being the majority group. It is vital to note these historic categories are social constructs and cultural and social associations may underlie differences. We conducted a literature search utilizing specific criteria to yield neuropathology papers that addressed the demographics and neuropathologies of relevance, then collated the findings into this review. We reveal that while there has been much progress in neuropathological research involving Latinos/Hispanics and Black Americans in the past decade, no cohesive conclusions could be extrapolated from the existing data due to the dearth of minority participants and even smaller amount of information related to the heterogeneity within each minority group, especially Latinos/Hispanics. Furthermore, we reveal an even greater scarcity in neuropathological studies involving Asian Americans, also a very heterogeneous group. We hope the presented findings will illuminate the paucity of minority representation in not just neuropathological research but the field of clinical research overall and serve to inspire clinicians and researchers to help reduce the health disparities underrepresented groups in the US face.

## Introduction

Clinically, Alzheimer’s disease (AD) is defined as a type of dementia distinguished by neurodegeneration that results in memory loss and deterioration of cognitive functions^24, 38^. Neuropathologically, AD is defined by aggregations of the amyloid-β (Aβ) protein, in the form of Aβ plaques, and tau protein, in the form of neurofibrillary tangles (NFTs)^13, 25, 97^. In addition to Aβ plaques and NFTs, neuropathologies associated with vascular dementia can also be concomitant^57, 78, 101^. Vascular dementia is the second most common cause of dementia following AD and neuropathologically can manifest as infarcts and hemorrhages as well as other vascular pathologies such as cerebral amyloid angiopathy (CAA) and arteriolosclerosis^9, 17, 23, 63, 90, 101^. While there has been progress with understanding disease phenotype (for review see ^89^) and progress with therapies having evidence of targeting and reducing Aβ plaques in the brain, there have yet to be treatments that fully cure or stop the progression of the disease^20, 22^. Although great strides have been made to combat this disease, most studies have focused on select populations or cohorts, specifically composed of persons identifying as non-Hispanic whites (NHWs)^28, 43, 73, 97^.

The population of persons age 65 and over in the United States (US) has significantly increased in the past decade—as much as a 36% increase^2^. US ethnoracial minorities made up 20% of this age demographic at the beginning of the decade, and increased to 24% by the end of it^2^. The NHW population of age 65 and over is projected to increase 29% by 2040 in comparison to the 115% increase of the ethnoracial minority population^2^. The largest US minority population is Latinos/Hispanics, followed by Black Americans, then Asian Americans, which are the fastest growing demographic^18, 53^. Other notable underrepresented groups are American Indians (Native Americans), Native Hawaiians, and Pacific Islanders^4^. It is important to note in this paper we will utilize historic terms (such as those within the US census) and these categories are social constructs and cultural and social associations may underlie differences. Diversity in studies maximizes variability in risk and protective factors. This can aid in studying clinically relevant transitions across the spectrum of a disease with the goal of identifying modifiable pathways to support maintenance of normal functions. As racial/ethnic groups in the US continue to grow in addition to longer life expectancies, more diverse elderly individuals will be seen for diagnosis and treatment of neurodegenerative diseases; thus, more research reflecting the population diversity is needed so that prevention, treatment, and prognosis strategies encompass all who are affected by the devastating impact of dementia.

### Heterogeneity of race and ethnic categories

Race and ethnicity are two terms fundamentally distinct from one another. Race historically has been used to describe the physical traits of an individual such as their eyes, hair, and skin, whereas ethnicity has been used to describe an individual’s cultural identity^74^. It is essential to remember these terms are socially constructed and hold no bearing on an individual’s biology. The categorizations for race and ethnicity in the US have evolved over time as self-identification shifts, immigration, and mixed racial heritage became more prevalent^1^.The term “race” has regretfully implied a sole focus on an individual’s morphology, and historically has not accounted for other background variables such as geographic origin, environmental factors, and sociocultural characteristics that influence these differences^43, 98^. While the historic terms employed in this review are not optimal given how restrictive and tentative they are, the U.S. Census uses them, which many neuropathology studies have utilized as well, and thus are presented as such in this review. Thankfully, there is a growing awareness that these historic terms alone do not aptly stratify these individuals in a scientific context and new approaches are being encouraged^1, 6, 43, 49^.

Furthermore, it is important to recognize there is heterogeneity within racial/ethnic categories. The terms Latinos and Hispanics are sometimes used interchangeably^65^. The term Hispanics historically has been used to describe those who are from Spain or other Spanish-speaking country, while Latino signifies those who originate from Latin America, regardless of their spoken language^62^. Moreover, it is noted the term Caribbean Hispanics is used to describe the Latino population that resides in Puerto Rico, Dominican Republic, and Cuba^96^. To further expand on distinctions within the Latino/Hispanic population of the US, some studies have partitioned this ethnicity based on area of decedents’ self-reported origin: Mexican, South American, Central American, and Caribbean^99, 100^. These categories are standardized by the US Census; hence studies have followed the same format^64^. The Mexican descendent population is the largest Latino/Hispanic group in the US and is spread out through the country, with more density in the Southwest^27^. Individuals from South and Central America also follow this distribution pattern, whereas Caribbean Latinos/Hispanics are more concentrated in eastern states^3^. These geographical distributions when examining AD cohorts can be immensely important as there have been reported socioeconomic and cultural differences within these groups that are associated with risk factors for AD^77^.

There is also heterogeneity within the Black American population. The largest subgroup within the US Black population is single-race non-Hispanic, comprising of 87% of the total US Black population^95^. The following largest is the multiracial non-Hispanic population, constituting 8% of the overall US Black population, with Black Hispanics making up the remainder 5%^95^. The majority of Black Americans are of West/Central African and European heritage, and some also have Native American roots^34^. An estimated 90% of the total Black population was born in the US in which most are descendants of enslaved people^84, 94^. Despite the Great Migration where the Black population had dispersed to areas in the US away from the South, the distribution in the South has begun to grow in the past few decades^92^. Another observed migration pattern in the last few decades has been a substantial increase among the foreign-born US Black population, making up 10% of the current US Black population in which the majority of foreign-born US Black persons, 88%, were born in African or Caribbean nations^94^.

Similarly, there is immense diversity within the Asian American population. There are 21 distinct Asian subgroups living in the US according to gathered data from the Census American Community Survey, with the largest being Chinese (including Taiwanese), followed by Asian Indian, Filipino, Vietnamese, Korean, and Japanese^15, 16^. Almost half (45%) of Asian Americans live in the western US, with nearly one third of the nation’s population living in California alone (30%), while 24% reside in southern states, 19% in the northeast, and 12% in the midwest^15^. Data have shown a substantial proportion of Chinese, Filipino, Vietnamese, Korean, and Japanese inhabit the western US, amounting to at least 43% distribution across all groups^46^. On the other hand, Asian Indians occupied the northeastern and southern regions of the US more frequently, accounting for over 29% distribution for each region^46^. By addressing the geographic, ethnic, and cultural variations that exist among race and ethnic subgroups, AD research can be refined to yield more precise methodology and analyses.

### Differences in clinical AD based on race and ethnicity

Differences in the prevalence and incidence of clinically defined AD and related dementias (ADRD) have been observed when comparing across race/ethnicity. AD prevalence is highest in Black Americans and Latinos/Hispanics, followed by NHWs, and then Asian Americans^21, 33, 40, 41, 61, 66-68, 70, 72, 79, 86, 96^. Notably, one study showed US-born NHWs, Hispanics, and other uncategorized races had lower frequency of dementia compared to their immigrant counterparts, except for non-Hispanic Blacks (NHBs), where it was an inverse correlation^72^. Studies have also shown differences in dementia prevalence between Latino subgroups. One study revealed a 4.8% prevalence of dementia among Mexican Americans ≥65 years residing in Sacramento County, California, with up to 31% prevalence in those aged 85 years or older^42^. In contrast, a study of Caribbean Hispanics residing in North Manhattan estimated a dementia prevalence for individuals aged 65-74 of 7.5%, 27.9% for those 75-84, and 62.9% for those 85 and older^41^. Latinos/Hispanics have been shown to have an earlier age of onset of AD when compared to NHWs and Black Americans, though the difference was marginal for the latter in some cohorts^21, 33, 42, 61, 72^. Both Latinos/Hispanics and Black Americans have a high prevalence of cardiovascular risk factors associated with ADRD, while prevalence of these risk factors is lower among NHWs followed by Asian Americans^40, 42, 61, 67, 96, 102^. In a California study that examined dementia incidence in Asian American subgroups, Filipino Americans had the highest incidence rate at 17.3 per 1000 person-years, and South Asian Americans (i.e., Asian Indian, Pakistani, Bangladeshi, Sri Lankan, or Nepalese) had the lowest rate at 12.1 per 1000 person-years^67^.

While many observable trends have been correlated with AD from a clinical perspective across different race/ethnicities, they do not confirm the presence of the hallmark protein aggregates that are currently are the gold standard for diagnoses of ADRD, in other words, the neuropathology. Do the neuropathological profiles of individuals with ADRD of the three largest minority groups in the US differ, as there have been noted cultural and geographic differences in these groups? Neuropathology studies have been conducted on predominantly NHW cohorts, so little is known about ADRD in these other racial/ethnic groups^6, 28, 43, 73^. For this review, we will discuss the current landscape of neuropathological findings in AD and vascular dementia in Latinos/Hispanics, Black Americans, and Asian Americans. We seek a more comprehensive understanding of the disease profile in these groups of individuals to provide improved diagnoses and develop effective countermeasure therapies or methods to allay the risk of AD and vascular dementia.

## Methods

### Inclusion and exclusion criteria

All literature in this review focused on signature pathologies associated with AD and vascular dementia in Latino/Hispanic, Black American, and Asian American cohorts. Studies meeting the inclusion criteria were published 1995 and onwards, peer-reviewed, specifically presented AD or vascular dementia neuropathological findings, and were conducted in the US. Neuropathologic evaluations of interest included but were not limited to Braak neurofibrillary tangle (NFT) stage, Consortium to Establish a Registry for Alzheimer’s Disease (CERAD) neuritic plaque score; Thal amyloid phase; and National Institute of Aging and Alzheimer’s Association (NIA-AA) criteria of overall AD diagnosis based on neuropathologic changes^13, 48, 71, 97^. For vascular dementia, pathologies of interest included, but were not limited to, infarcts, hemorrhages, arteriolosclerosis, atherosclerosis, and CAA. As for staging schemes and creating a consensus for diagnosing vascular dementia, there have been multiple attempts^23, 55, 83, 90, 92^; however, there is no universally used system in place. Exclusion criteria included papers that contained only living cohorts (i.e., cohorts not having neuropathologic evaluation of persons after death) presented data with no mention of AD neuropathologies specifically (e.g., genotyping, neuropsychological tests, etc.), studies that were not conducted on human subjects, and articles that were not sourced from peer-reviewed journals.

### Search strategy

The literature review was conducted by searching specific MeSH terms in PubMed, Scopus, and Web of Science to yield the peer-reviewed articles investigating AD and vascular neuropathology among Latinos/Hispanics, African Americans/Black Americans, and Asian Americans on November 19, 2021. The MeSH search term refinement process for these databases was guided by a UC Davis Health medical librarian. The full list of search terms can be found in the supplemental methods at the end of this review.

## Results

Neuropathologic findings on US minority groups were often compared to NHWs. For the purposes of this review, we will use Latinos/Hispanics as the collective term to describe this demographic unless a mentioned study further partitions out subgroups, in which the terms that are consistent with the study will be used. This same principle applies to the term Black Americans being used for mentions of this group unless the study denotes otherwise. Details of selected main papers are within Table 1 relating to neuropathological data of Latinos/Hispanics and/or Black Americans as many of these studies compared these groups. Additional studies on Asian Americans are in Table 2.

**Table 1:** **Literature with neuropathological data of Latinos/Hispanics and/or Black Americans.** For the ethnicity/race column, the mentioned terms used in each respective paper stated are followed with the universal synonym in parentheses to provide consistency.

**Citation (PMID)**	Cohort and location*	**Numbers of Ethnicity/Race(s) examined**	**Total cohort size**	**Pathologies examined**	**Inclusion/Exclusion Criteria**	**Main findings**
Sandberg G, …, Troncoso JC. Neurobiol Aging, 2001. (11182466)	Maryland ME office; study carried out at University of Maryland	☐ 58 African Americans (Black Americans) ☐ 80 Whites (NHWs)	138	☐ AD	Inclusion Neuropath consultations at Maryland ME office between 1990 to 1998 Age between 40-79 years Non-natural manner of death	No significance differences in prevalence of SP or NFT between groups.
Wilkins CH, …, Morris JC. Arch Neurol, 2006. (16401740).	Washington University ADRC; greater metropolitan St. Louis, MO	☐ 10 African Americans (Black Americans) ☐ 10 Whites (NHWs)	20	☐ AD ☐ CVD ☐ LBD	Inclusion Autopsy between 1990 to 2000 NHWs were matched to age (+/- 5 yrs of death, sex, and CDR at death Over 50 yrs of age at enrollment	No significant neuropathological differences were found in both groups across all pathologies examined. No group differences in the presence or number of infarctions, plaques, NFTs, Lewy bodies, CAA.
Riudavets MA, …, Troncoso JC. J Neuropathol Exp Neurol 2006. (17146288)	Maryland ME office; study carried out at University of Maryland	☐ 100 Blacks (Black Americans) ☐ 100 Whites (NHWs)	200	☐ AD ☐ CVD	Inclusion Aged 65 to 95 years at death Consecutive autopsies at Maryland ME office between 2002 to 2005	Race not a significant factor in frequency or severity of AD lesions (Amyloid Beta plaques and NFTs). No significant difference in vascular lesions by race. ApoE4 increased risk of AD lesions similarly in each race.
Mehta KM, ..., Miller BL. Neurology, 2008. (18003939)#	>30 ADCs; NACC database	☐ 1,301 Latinos (Hispanics) ☐ 3,563 African Americans (Black Americans) ☐ 451 Asian ☐ 162 American Indians ☐ 25,160 Whites (NHWs)	30,916 (3,017 with npath)	☐ AD ☐ CVD	Inclusion: Aged 65 yrs or older Dx of possible/probable AD Seen at an ADC between 1984-2005 Exclusion: Identified as other race Missing death data	African American and Latino/Hispanic patients had similar AD neuropathologies when compared to NHWs. Both Latinos and African Americans were equally likely to have Braak NFT stages V and VI. Neurovascular pathology and NP presence were more common in Latinos than NHW.
Ringman JM, ..., Vinters HV. JAMA Neurology, 2014. (24797962)*	>30 ADCs; NACC database	☐ Hispanic (Latinos) ☐ African Americans (Black Americans) ☐ Whites (NHWs)	425	☐ AD ☐ CVD	Inclusion Severe and no CAA Cognitive impaired and meeting NIA Reagan criteria for AD Exclusion Did not identify as Hispanic, African Americans or NHW	Hispanics with neuropathologically confirmed AD more likely to have severe CAA than non-Hispanics. African Americans did not differ significantly with NHWs.
Barnes LL, …, Schneider JA. Neurology, 2015. (26180136).	Rush University, ADRC Chicago, Illinois	☐ 41 Blacks (Black Americans) ☐ 81 Whites (NHWs)	122	☐ AD ☐ CVD ☐ LBD	Inclusion Consecutive autopsies, age, sex, education, and cognition matched NHWs to Black Americans ~2:1	Blacks with AD more likely to have mixed brain pathologies compared to NHWs with AD.
Graff-Radford NR, …, Dickson DW. Alz Dement, 2016. (27094726)	32 past/present ADCs; NACC database	☐ 110 African Americans (Black Americans) ☐ 2,500 White (NHWs)	2,610	☐ AD ☐ CVD ☐ LBD ☐ TDP/FTD	Inclusion Available NACC data from 2005 to 2015 Dementia at last clinic visit and went to autopsy Exclusion Participants reporting a race other than African Americans or NHWs AD neuropathologic change in January 2015; this data was excluded due to the small sample size with ADNC data to date	AD, LBD, and CVD more common in African Americans thank NHWs. African Americans had higher Braak NFT Stage and CERAD when compared to NHWS. African Americans had more CVD pathologies when compared to NHWs.
Kamara DM, …, Walker LC. J Alzheimers Dis, 2018. (29614657).	Emory ADRC Atlanta, GA	☐ 18 African Americans (Black Americans) ☐ 19 Caucasians (NHWs)	37	☐ AD ☐ CVD	Inclusion Autopsies between 2003 to 2014 End stage AD Groups matched as close as possible for age, disease duration, APOE type, sex, level of education, and post-mortem interval	No significant differences in CAA in person with AD between groups.
Soria JA, …, Rissman RA. J Alzheimers Dis, 2018 (30412501)	UCSD ADRC San Diego, California	☐ 53 Latinos (Hispanics)	53	☐ AD ☐ CVD ☐ LBD	Inclusion Older adults with Latino ethnicity Autopsied from 1991 to 2017 Exclusion Presenilin 1 mutation cases interval between last clinical encounter and death > 2.5 years Insulin-dependent diabetes, major stroke or neurological illness, or self-reported alcohol or drug abuse	Clinic dx of AD at last clinical evaluation had 97.1% sensitivity and 57.9% specificity against autopsy-verified AD in Latinos.
Filshtein TJ, …, DeCarli C. J Alzheimers Dis, 2019. (30775996)	University of California, Davis (UCD) ADC; Sacramento, CA	☐ 28 Hispanic (Latinos) ☐ 35 Black (Black Americans) ☐ 360 NHWs	423	☐ AD ☐ CVD	Inclusion: Dementia at last visit before death went to autopsy between 2000 and 2017	Hispanics had lower (14%) AD (non-mixed) than NHWs (43%) and Black (43%). Blacks and Hispanics had higher CVD (40% and 54% respectively) compared to NHWs (28%). Most common neuropath dx was AD across all groups: 80.5% in NHWs, 80% in Black, and 67.9% in Hispanics regardless of concomitant diagnosis.
Santos OA, ..., Murray ME. Alz Dement, 2019. (30792090)	Florida Autopsies Multi-Ethnic cohort (FLAME) State of Florida brain bank	☐ 67 Hispanic (Latinos) ☐ 19 African Americans (Black Americans) ☐ 1,539 Caucasian (NHWs)	2,809	☐ AD ☐ LBD	Inclusion: Brain tissue was received on or before August 2015 within state of Florida brain bank Autopsy confirmed AD cases regardless of clinical dx Exclusion Non-AD autopsy confirmed cases AD cases with known mutations	Thal amyloid phase did not differ across all groups. Hispanics were found to be twice as likely to have higher Braak NFT stage compared to NHWs. African Americans did not differ from NHWs for Braak NFT staging.
Weissburger GH, ..., Salmon DP. J. Alzheimers Dis., 2019. (30636736)	UCSD ADRC San Diego, California	☐ 14 Hispanic (Latinos) ☐ 20 NHWs	34	☐ AD ☐ CVD	Inclusion Persons with AD dementia who died and autopsied between 1989-2016 >=95 on DRS at 1^st^ clinical evaluation Exclusion Presenilin 1 mutations with early age of onset **Insulin-dependent diabetes, major stroke or neurological illness, or self-reported alcohol or drug abuse	Groups had similar overall AD pathology burden. Hispanics with AD had more small parenchymal arteriolar disease and CAA than NHW with AD. Groups did not differ in other aspects of cerebrovascular pathology such as infarctions and/or hemorrhages.

# for Mehta *et al.* numbers of Ethnicity/Race(s) examined represent deceased persons, only a subset of cases (n= 3,017) had neuropathology data--npath details of ethnoracial group were not listed. *for Ringman *et al.* data within paper not sufficient to derive numbers of Ethnicity/Race(s) examined with neuropathologic evaluations. ** previous UCSD ADRC cohort studies state this exclusion, although not stated directly within paper. Abbreviations: AD=Alzheimer’s disease, ADC/ADRC= Alzheimer’s Disease (research) Center, CAA=cerebral amyloid angiopathy, CVD=Cerebrovascular disease related pathologies, DLB=Dementia with Lewy Bodies, DRS=Dementia rating scale, dx=diagnosis, NACC=National Alzheimer’s Coordinating Center, ME= medical examiner, npath=neuropathology, LBD= Lewy body dementia, FTD=Frontotemporal dementia, NFT=neurofibrillary tangles, NHW=Non-Hispanic Whites, UCSD=University of California San Diego. *Cohort name was listed if given and location includes medical center/institution/data source.

### AD and vascular dementia neuropathology in Latino/Hispanics

One observed neuropathological trend in Latinos/Hispanics was cerebrovascular pathologies, such as infarcts, CAA, arteriolosclerosis, and atherosclerosis, that were typically associated with dementia and/or AD diagnoses (See Table 1). A 2010 study using the National Alzheimer’s Coordinating Center (NACC) database showed Latinos/Hispanics were more likely to have neurovascular pathology compared to NHWs^70^. In persons with dementia during life, studies conducted at the Alzheimer’s Disease Research Centers (ADRCs) at both University of California, Davis (UCD) and the University of California, San Diego (UCSD) reported a higher frequency of concomitant neurovascular pathologies compared to NHWs^29, 102^. One comparison of interest between these studies with respect to cerebrovascular disease (CVD) pathologies—specifically microinfarcts and macroinfarcts—is Filshtein *et al.* found occurrence was higher in Latinos/Hispanics compared to NHWs in the UCD cohort^29^, while Weissburger *et al.* discovered there were no significant differences between the two groups in their UCSD cohort^102^. This contradiction may be because persons with evidence of *in-vivo* hemorrhages, strokes, and other major agonal infarcts were excluded from the UCSD study^102^. A study also using the National Alzheimer’s Coordinating Center database illustrated this theme in the specific context of CAA, in which Latino individuals with neuropathologically confirmed AD were more probable to have severe CAA than NHWs^81^. The study done by Weissburger *et al.* also supported this trend^102^. Another study by UCSD further validated this pattern by finding higher CAA burden in the AD group compared to the no pathology group, which was defined as not having significant brain pathologies, and the non-AD pathology group was defined as only have tauopathies, frontotemporal dementia (FTD), progressive supranuclear palsy (PSP), dementia with Lewy bodies (DLB), or Parkinson’s Disease (PD) with neocortical Lewy bodies, in an all-Latino cohort^91^.

As for hallmark AD pathologies, Aβ plaques and NFTs, there were more inconsistent patterns between Latinos/Hispanics and NHWs. The study done by Filshtein* et al.* utilizing demented cases from the Alzheimer’s Disease Center at UCD, a California based cohort, revealed Latinos/Hispanics had the lowest occurrence of AD clinicopathological diagnosis without the involvement of CVD, including lower frequencies of persons at higher Braak NFT stage compared to NHWs and Black Americans^29^. This is consistent with literature where Latino cohorts tended to exhibit concomitant neurovascular pathologies with their AD diagnoses^29, 102^. Conversely, Santos *et al.,* in a Florida based cohort, demonstrated Latinos/Hispanics were twice as likely to have a higher Braak NFT stage than NHWs^86^, while a study conducted by Mehta *et al.,* including cases with a clinical possible/probable diagnoses of AD, revealed Braak NFT stage did not differ significantly between Latinos/Hispanics and NHWs^70^. The study by Weissburger* et al.* also showed both groups (NHWs and Latinos/Hispanics) had similar Braak NFT stage^102^. Regarding plaques, which can include neuritic plaques (amyloid plaques containing dystrophic neurites) in some literature, Mehta *et al.* found neuritic plaques were more frequent in Latinos/Hispanics compared to NHWs^70^. However, the results by Filshtein *et al.* revealed Latinos/Hispanics had the lowest proportion of CERAD frequent neuritic plaque score, implying that neuritic plaques may not be as much of a major contributing pathology to their dementia^29^. Santos *et al.*, excluding persons that did not have autopsy confirmed AD and cases with known mutations, opted to use Thal amyloid phase to categorize plaque presence, in which the phases did not differ between Latinos/Hispanics and NHWs^86^. Notably, Latino/Hispanic participants in these studies may represent diverse ethnic groups in terms of geography and nation of origin leading to seemingly contradictory findings. For instance, Santos *et al.* had utilized the Florida Autopsies Multi-Ethnic (FLAME) cohort located at the Mayo Clinic of Florida for their study, which consisted of individuals primarily from the Caribbean origin for their Latino group, whereas Weissburger *et al.* and Soria *et al.* had utilized cohorts from the UCSD ADRC, which comprised of individuals primarily of Mexican descent for their Latino/Hispanic group^86, 91, 102^. Furthermore, these studies also had slightly different inclusion and exclusion criteria, as outlined in Table 1, that may also contribute to discrepancies.

### AD and vascular dementia neuropathology in Black Americans

Like Latinos/Hispanics, cerebrovascular pathologies are also commonly observed in Black Americans, but the pattern is not completely consistent which may be due to cohort inclusion/exclusion criteria, demographic locations, and recruitment strategies. A study at the Rush Alzheimer’s Disease Clinical Core, based in the Chicago Illinois area, revealed Black decedents had significantly greater severity in both atherosclerosis and arteriolosclerosis when compared to NHWs^7^. The study by Filshtein *et al.* corroborates this finding, in which their results of persons with dementia demonstrated Black participants had a higher proportion of CVD compared to NHWs^29^. Another study utilizing patient data from over 30 Alzheimer’s disease centers across the country also found Black Americans were more likely to have had a contributing diagnosis of vascular dementia than NHWs, although this study was based on small group numbers and did not account for center biases^40^. Interestingly, Mehta *et al.* revealed Black Americans had similar neurovascular pathology rates as NHWs on autopsy, in contrast to the consensus of the other studies^70^. Likewise, the results from a study on a cohort based in Washington University’s Alzheimer’s Disease Research Center (ADRC) also showed no differences in cerebrovascular infarcts between the NHW and Black American participants^107^. Multiple studies found CAA burden did not differ significantly between Black Americans and NHWs^56, 81, 82^. In contrast, Graff-Radford *et al.* observed Black Americans had significantly greater frequencies of CAA in addition to the other vascular neuropathologies (i.e., infarcts, hemorrhages, arteriolosclerosis, atherosclerosis) in comparison to NHWs^40^; however this study utilized data from multiple cohorts and did not control for center biases.

For AD pathologies, there were also contradictions in the literature. Findings from Barnes *et al.* revealed Black decedents were less likely to have AD-only pathology, defined by neuritic plaques and NFTs as the single contributing pathology to their dementia diagnosis compared to NHW decedents^7^. Along similar conclusions, Filshtein *et al.* demonstrated mixed pathologies were more common in Black decedents than in NHW decedents^29^. With respect to hallmark AD proteinopathy comparisons, such as neuritic and diffuse plaque counts, Thal amyloid phase, and likelihood of higher Braak NFT stage, multiple studies showed no significant neuropathological differences in both categories for either patient demographic^82, 85, 86, 107^. In contrast, more than one study demonstrated Black American decedents were more likely to exhibit higher Braak NFT stage^29, 40, 70^. Graff-Radford *et al.* also indicated Black American participants had greater CERAD-frequent scores for neuritic and diffuse plaques^40^, whereas Mehta *et al.* revealed Black Americans had similar neuritic and diffuse plaque counts as NHWs^70^. Both studies had utilized data from the NACC database, but this conflict in findings may be due to the fact the Mehta *et al.* study had a larger sample size from the longitudinal window from 1984 through 2005^70^, compared to the Graff-Radford *et al.* study which recruited data from a smaller sample size from 2005 to 2015^40^. As with the previous section, discrepancies may lie within cohort selection criteria as highlighted in Table 1.

### AD and vascular dementia neuropathology in Asian Americans

AD and vascular dementia pathological trends for Asian Americans compared to NHWs are largely unexplored for all pathological categories as literature for this minority group in this specific context is still sparse. Of the studies conducted, most have focused on Japanese Americans, specifically men, through the Honolulu Asian Aging Study (HAAS) which includes very few, if any other subgroups. The HAAS was established in 1991 and comprised surviving participants of the Honolulu Heart Program, a prospective, community-based cohort study of heart disease and stroke established in 1965^36, 54, 93, 105^. For neurovascular pathologies, a study revealed microinfarcts were significantly more common in Japanese American men in HAAS compared to Caucasian women in the Nun Study (NS)^106^. Another finding from the HAAS showed that the frequency of microvascular lesions as the contributing dementia pathology was nearly the same as AD pathologies^104^; however, a later paper relative to this one showed that microvascular infarcts as the dominant or exclusive contributing lesion to dementia were the most frequent among decedents, then followed by AD lesions^103^. There were additional findings on the HAAS revealing dementia frequency increased with neuritic plaques in decedents with NFTs and even further with CVD lesions^76^. Interestingly, one analysis showed microinfarcts were strongly associated with poor cognitive function score in non-demented individuals, whereas NFTs were strongly associated with poor cognitive function score in demented individuals^59^. For neuropathologic change involving AD proteinopathies, a more recent study showed that the HAAS was more resistant to Aβ accumulation, but the NS was more resistant to neurofibrillary degeneration for individuals without Aβ accumulation^58^.

**Table 2:** **Literature of neuropathological data of Asian Americans.** For the ethnicity/race column, the mentioned terms used in each respective paper stated are followed with the universal synonym in parentheses to provide consistency.

**Citation (PMID)**	Cohort and location*	**Numbers of Ethnicity/Race(s) examined**	**Cohort size**	**Pathologies examined**	**Inclusion Criteria**	**Main findings**
White L, …, Markesbery W. Ann N Y Acad Sci, 2002. (12480729)	HAAS, Oahu, HI	285 Japanese American men (Asian Americans)	285	☐ AD ☐ CVD ☐ LBD	Came to autopsy between 1991 to 1999	CVD as an explanation for dementia nearly equal to AD. Most essential and inclusively related CVD lesion to dementia was multiple microinfarctions. Among the 27 decedents with high levels of microvascular lesions and no other lesions, 70% were demented. In 33% of subjects, demented could not be contributed to AD, CVD, Hpscl and/or LBD.
Petrovitch H, …, White LR. Ann Neurol 2005. (15562458)	HAAS, Oahu, HI	333 Japanese American men (Asian Americans)	333	☐ AD ☐ CVD	Came to autopsy between 1992 to 2001 Cognitive function testing within 4 years of death	Among dementia cases, 24% were linked to CVD. Dementia frequency increased in men with NFTs and increasing NP density, increasing further with CVD presence. 20% of persons with neocortical NFT without NP. 9% of cases had no NP, NFT or CVD.
White L. J Alzheimers Dis. 2009. (19661625)	HAAS, Oahu, HI	443 Japanese American men (Asian Americans)	443	☐ AD ☐ CVD ☐ LBD	Came to autopsy between 1992 to 2004	Microvascular infarcts were most frequently the sole or dominant lesion found in demented decedents. AD was the second most dominant contributing lesion to demented decedents. Co-dominant lesions (usually microvascular infarcts and AD) were the third common contributor to dementia in decedents.
Launer LJ, …White LR Ann Neurol 2011. (22162060)	HAAS, Oahu, HI	436 Japanese American men (Asian Americans)	436	☐ AD ☐ CVD	Came to autopsy between 1992 and 2001	Significant association of higher numbers of microinfarcts with lower brain weight and with poorer antemortem global cognitive scores in those with no dementia. NFTs were associated with brain weight, especially in demented individuals.
White LR, …, Montine TJ; Neurology, 2016. (26888993)	HAAS, Oahu, HI and the Nun Study (NS) from Montreal, Canada	334 Japanese American men (Asian Americans) 774 Caucasian women (NHWs)	1,108	☐ AD ☐ CVD ☐ LBD		Microinfarcts are more prevalent in Japanese American men than Caucasian women. AD pathological changes and neocortical LBs more frequent in NHW women. Total burden of comorbid pathologies was most relevant in determination of cognitive impairment in both cohorts.
Latimer CS, … Montine TJ. J Neuropathol Exp Neurol, 2017. (28499012)	HAAS, Oahu, HI and the Nun Study (NS) Montreal, Canada	762 Japanese American men (Asian Americans) 500 Caucasian women (NHWs)	1,262	☐ AD ☐ CVD ☐ LBD	Completed NIA-AA ABC scores	Both cohorts were most resistant to neuritic plaque accumulation and least resistant to neurofibrillary degeneration. Significant differences in prevalence of AD, CVD, and LBD between these 2 cohorts. HAAS (Japanese American men) had higher prevalence of high level neuropathologic change of AD and vascular brain injury (VBI) compared to the NS (Caucasian women), which had high neuropathologic change of LBD.

All published studies within table included individuals from the Honolulu Asian Aging Study (HAAS) which is a continuation of the Honolulu Heart Program^54^. Persons within HAAS consisted of Japanese American men born between 1900 and 1920 living in Oahu during baseline examination in 1965. Participants were evaluated for dementia starting in 1991 (age 73 to 94 years), the autopsy program began in 1992. Abbreviations: AD=Alzheimer’s Disease, CVD=Cerebrovascular disease, Hpscl=Hippocampal Sclerosis, LBD=Lewy body disease, NP=neuritic plaques, NFT=Neurofibrillary Tangle

### Native American, Alaska Native, Native Hawaiian, other groups, and points of further research

**Figure 1:**
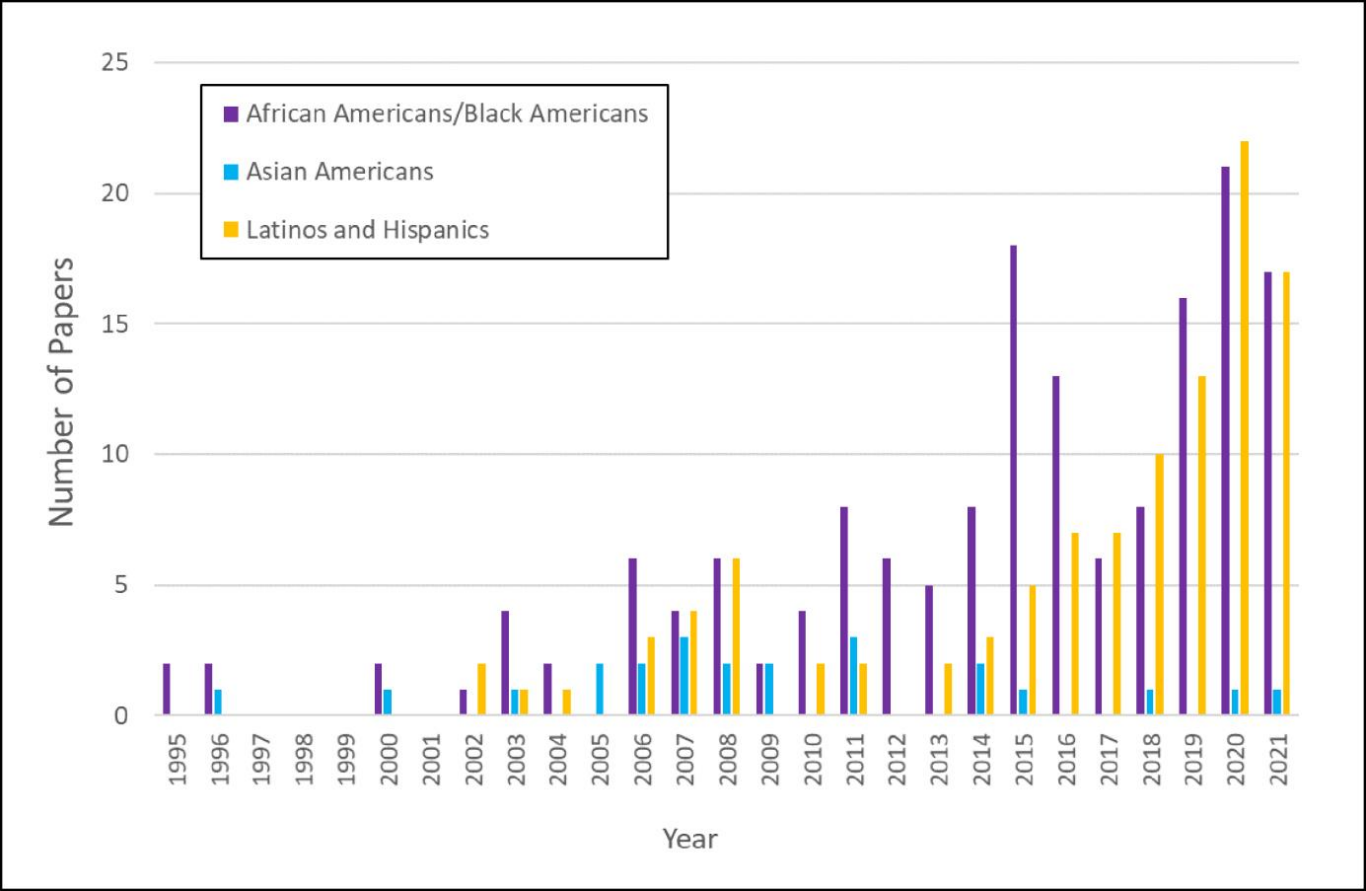
Number of papers found on PubMed as of November 19, 2021 by year using the search terms “Alzheimer’s brain pathology” along with the demographic term (categories in legend, see supplemental section for further information).

The scope of this paper had focused on the neuropathology of the three largest minority groups of the US, as those were the demographics that offered adequate findings to collate into a cohesive and purposeful review. The current presented literature offers a foundation for AD and vascular dementia research in underrepresented US groups and seems to only expand each year (see Figure 1), with more research being conducted on a greater variety of cohorts and sites. The existing findings are concentrated and substantial enough to serve as preliminary data for comparison of future findings, depending on the objective demographic. Nonetheless, despite the upward trends of more AD and vascular dementia research being conducted in diverse cohorts, there are still many gaps that need to be filled and other demographics that need to be considered. For example, Alaskan Natives and American Indians (Native Americans) constitute the fourth largest distinct (i.e. one race) population of the US^4^, yet there is a paucity of medical studies on persons of these backgrounds. This may be due to cultural aspects, where those who valued tradition (including religious beliefs) strongly advocated for the body to buried whole^52^. This dearth of information also applies to Native Hawaiians and other Pacific Islanders, despite being the fifth largest single race population and second fastest growing race in the country behind Asian Americans^4, 45^. The lack of neuropathology literature that captures the diversity of Asian Americans also highlights imbalances in research, as it is most probable the neuropathological trends of Japanese Americans would not accurately encompass the depth and breadth of diversity of persons across the Asian continent. An overall paucity of literature presently exists in comprehensive studies centering on these mentioned groups and is not limited to specifically neuropathology studies.

While there has been advances in neuropathology literature focused on Latinos/Hispanics and Black Americans in recent years compared to other minority groups, there are still limited participants in these cohorts. The existing literature is limited in sample size, which varies widely between studies and typically with the minority groups representing a small fraction of the cohort (see Tables 1 and 2). Furthermore, studies can have certain inclusion and exclusion criteria that may hinder participation in select groups; for example, exclusion of CVD for AD studies may decrease frequencies of certain minority groups with higher frequencies of CVD^50^.

These constraints from low minority group recruitment may be due to numerous factors, including lack of access to healthcare, historical abuses of minority groups for medical research, mistrust of the healthcare system making participants less likely to agree to participate in clinical trials or autopsy programs, and language barriers^5, 8, 10, 11, 30, 31, 37, 49, 75, 100^. With respect to retention, a systematic review highlights a lack of literature that examines retention exclusively from recruitment^39^. Socioeconomic circumstance was shown to be the most powerful contributor to the absence of participants for longitudinal studies involving ethnoracial minorities^26^. Low socioeconomic status largely impacts access to health care resources such as regular visits to a health professional as a result of being uninsured^10, 87, 88^, in which patients may not only lack the direct care they need but also the general awareness of clinical study enrollment opportunities. Flexible scheduling played a substantial role in participation as many individuals were restrained by work or childcare obligations for their appointments as well as transportation^26, 32, 69^. Financial compensation was a major influencer in recruitment amongst ethnoracial minorities^51, 69^; it has been reported that members of the Latino/Hispanic community were motivated by monetary compensation for their time because they experienced economic hardships^69^.

Patients may also feel discrimination in the process of seeking care, especially among non-Whites for their race, color, and/or ethnicity^35^. Half of Black Americans report they have faced healthcare discrimination, and one third of Asian Americans and Latino Americans similarly report having experienced healthcare discrimination as well^35^. A vast majority of non-White Americans believe that in the importance of having AD and dementia care providers to understand their ethnic/racial backgrounds, such as Native Americans, Black Americans, Latinos/Hispanics, and Asian Americans^35^. However, less than half of Black and Native Americans are confident there are culturally competent providers, and only roughly 3 in 5 Asian Americans and Latinos/Hispanics are confident^35^. A few studies have attempted to understand barriers and willingness for brain donation across major US racial ethnic groups: NHWs, Latinos/Hispanics, Black Americans, and Asian Americans^11, 12^. While conducting focus groups, the first study revealed concerns, attitudes and beliefs around brain donation that fell into three categories: 1) religious beliefs 2) concerns and misconceptions about brain research and 3) the role of the family^7^. A follow up study surveying NHWs as well as 169 African Americans, 50 Asians, and 61 Hispanics revealed older age, Latino ethnicity and understanding of brain use by researchers and what participants need to do to ensure brain is donated were positive predictive factors, while the belief that the body should remain whole at burial, African/African American race, and concern researchers might not be respectful of the body during autopsy were negative predictive factors^11, 12^. The belief that the body should remain whole was shared amongst Latinos/Hispanics, African Americans, and Asian Americans, which was a similar sentiment of Alaskan Natives and American Indians mentioned earlier^52^. This further illustrates the substantial role cultural barriers may play in cohort participation from US minority groups.

Knowledge, stigma, and apprehension of ADRD also differ across ethnic/racial groups. For example, one study revealed that NHWs tended to have greater knowledge about AD compared to Black Americans, and Black Americans had same or greater levels of concern about getting AD as NHWs depending on their geographic location^47^. Another study discovered concern about developing ADRD in Native Americans, Black Americans, and Latinos/Hispanics is noticeably lower compared to NHWs^35^, which contradicts the finding about Black Americans in the aforementioned study, possibly due to region differences where the data was taken. It has also been shown that Asian Americans do not exhibit a strong concern of ADRD as many believed it was a natural occurrence for aging people^14^. Multiple papers have denoted that Asian Americans had beliefs of stigma of persons with AD, which played a significant role in seeking care from primary care providers for AD^14, 19, 60^. Limited knowledge on not only ADRD but also the brain removal process poses some hesitance on minority subject participation^8, 11^. As stated above, some themes that subjects or family members of subjects shared skepticism on were understanding the purpose of studying a decedent’s brain, misconceptions on how the brain is used or collected for research, and overall knowledge about the brain donation procedure^11^.

It is important to recognize the existing inadequacies and confines of the study recruitment process for US minority groups to further advance the representation of these populations in biomedical research. Fortunately, there has been progress to minimize these barriers. The UC Davis ADC utilized many avenues to increase diversity in enrollment in research cohorts, such as satellite clinic sites, increasing face to face screening at community events, options of in-home visits, compensation for transportation to clinic visits, dedicated drivers to transport participants to visits, and employing bicultural and bilingual individuals with proficiency of the involved populations^44^. These methods facilitated a substantial increase in the number of ethnic minority participants, as much as a four-fold increase^44^; this approach also led to more diversity in other variables as well, such as educational background^44^. A later study showed that mailing recruitment letters was the most successful method in a multi-modal recruitment approach in enrolling more ethnoracial minorities for ADRD cohorts^80^. As these issues get addressed on a more widespread scale, significant advancements can be made not only in the field of neuropathology, but all fields of clinical research.

## Acknowledgements

This work was supported by the National Institute on Aging of the National Institutes of Health under Award Numbers AG062517, AG052132, AG050782 and AG056519, and supported by the California Department of Public Health Alzheimer’s Disease Program (Grant # 19-10611) with partial funding from the 2019 California Budget Act. The views and opinions expressed in this manuscript are those of the author and do not necessarily reflect the official policy or position of any public health agency of California or of the United States government.

## Supplementary Material

Additional resources and electronic supplementary material: supplementary material
