# Supplementary material for: Neuropathology studies of dementia in US persons other than non-Hispanic whites
Source: Free Neuropathol. 2022 Mar 10;3:6. doi: 10.17879/freeneuropathology-2022-3795 (PMC9007571; doi:10.17879/freeneuropathology-2022-3795)
Supplement: Supplementary file 1 [file freeneuropathol-03-06-3795-s1.pdf]

Free Neuropathology 3:6 (2022)
doi: https://doi.org/10.17879/freeneuropathology-2022-3795

Supplemental methods

Latino demographic search terms

- "Alzheimer's" "neuropathology" "Latino"
- "Alzheimer's" "neuropathology" "Hispanic"
- "Alzheimer's" "brain pathology" "Latino"
- "Alzheimer's" "brain pathology" "Hispanic"
- "dementia" "neuropathology" "Latino"
- "dementia" "neuropathology" "Hispanic"
- "dementia" "brain pathology" "Latino"
- "dementia" "brain pathology" "Hispanic"
- "vascular dementia" "pathology" "Latino"
- "vascular dementia" "pathology" "Hispanic"

Black American demographic search terms

- "Alzheimer's" "neuropathology" "African American"
- "Alzheimer's" "brain pathology" "African American"
- "Alzheimer's" "neuropathology" "Black American"
- "Alzheimer's" "brain pathology" "Black American"
- "dementia" "neuropathology" "African American"
- "dementia" "neuropathology" "Black American"
- "dementia" "brain pathology" "African American"
- "dementia" "brain pathology" "Black American"
- "vascular dementia" "pathology" "African American"
- "vascular dementia" "pathology" "Black American"

Asian American demographic search terms

- "Alzheimer's" "neuropathology" "Asian American"
- "Alzheimer's" "brain pathology" "Asian American"
- "dementia" "neuropathology" "Asian American"
- "dementia" "brain pathology" "Asian American"
- "vascular dementia" "pathology" "Asian American"

Copyright: © 2022 The author(s). This is an open access article distributed under the terms of the Creative Commons Attribution 4.0 International License (https://creativecommons.org/licenses/by/4.0/), which permits unrestricted use, distribution, and reproduction in any medium, provided the original author and source are credited, a link to the Creative Commons license is provided, and any changes are indicated. The Creative Commons Public Domain Dedication waiver (https://creativecommons.org/publicdomain/zero/1.0/) applies to the data made available in this article, unless otherwise stated.
